# Utility of whole exome sequencing for the early diagnosis of pediatric-onset cerebellar atrophy associated with developmental delay in an inbred population

**DOI:** 10.1186/s13023-016-0436-9

**Published:** 2016-05-04

**Authors:** Hisham Megahed, Michaël Nicouleau, Giulia Barcia, Daniel Medina-Cano, Karine Siquier-Pernet, Christine Bole-Feysot, Mélanie Parisot, Cécile Masson, Patrick Nitschké, Marlène Rio, Nadia Bahi-Buisson, Isabelle Desguerre, Arnold Munnich, Nathalie Boddaert, Laurence Colleaux, Vincent Cantagrel

**Affiliations:** Clinical Genetics Department, Human Genetics and Genome Research Division, National Research Center, Cairo, 12311 Egypt; INSERM UMR 1163, Laboratory of Molecular and Pathophysiological Bases of Cognitive Disorders, Paris, France; Paris Descartes – Sorbonne Paris Cité University, Imagine Institute, Paris, France; Genomic Platform, INSERM UMR 1163, Paris Descartes – Sorbonne Paris Citée University, Imagine Institute, 75015 Paris, France; Bioinformatic Platform, INSERM UMR 1163, Paris Descartes – Sorbonne Paris Citée University, Imagine Institute, 75015 Paris, France; Imagine Institute, INSERM UMR 1163, Genetics of mitochondrial diseases, 75015 Paris, France; Imagine Institute, INSERM UMR 1163, Embryology and genetics of human malformation, 75015 Paris, France; Pediatric Neurology, Necker Enfants Malades University Hospital, APHP, 75015 Paris, France; Department of Genetics, Necker Enfants Malades University Hospital, APHP, 75015 Paris, France; Department of Pediatric Radiology, Necker Enfants Malades University Hospital, APHP, 75015 Paris, France

**Keywords:** Cerebellum atrophy, Intellectual disability, Exome sequencing, Molybdenum cofactor deficiency, MOCS2, KIF1A

## Abstract

**Background:**

Cerebellar atrophy and developmental delay are commonly associated features in large numbers of genetic diseases that frequently also include epilepsy. These defects are highly heterogeneous on both the genetic and clinical levels. Patients with these signs also typically present with non-specific neuroimaging results that can help prioritize further investigation but don’t suggest a specific molecular diagnosis.

**Methods:**

To genetically explore a cohort of 18 Egyptian families with undiagnosed cerebellar atrophy identified on MRI, we sequenced probands and some non-affected family members via high-coverage whole exome sequencing (WES; >97 % of the exome covered at least by 30x). Patients were mostly from consanguineous families, either sporadic or multiplex. We analyzed WES data and filtered variants according to dominant and recessive inheritance models.

**Results:**

We successfully identified disease-causing mutations in half of the families screened (9/18). These mutations are located in seven different genes, *PLA2G6* being the gene most frequently mutated (*n* = 3). We also identified a recurrent de novo mutation in the *KIF1A* gene and a molybdenum cofactor deficiency caused by the loss of the start codon in the MOCS2A open-reading frame in a mildly affected subject.

**Conclusions:**

This study illustrates the necessity of screening for dominant mutations in WES data from consanguineous families. Our identification of a patient with a mild and improving phenotype carrying a previously characterized severe loss of function mutation also broadens the clinical spectrum associated with molybdenum cofactor deficiency.

**Electronic supplementary material:**

The online version of this article (doi:10.1186/s13023-016-0436-9) contains supplementary material, which is available to authorized users.

## Background

Atrophy and hypoplasia of the cerebellum are neuroradiological findings identified in pediatric-onset cerebellar ataxias and generally associated with imbalance, poor coordination and developmental delay. Hereditary cerebellar atrophy in childhood is a clinically and genetically heterogeneous group of conditions. They include a vast number of differential diagnoses with overlapping clinical findings such as intellectual disability and epilepsy [1, 2].

The specificity of cerebellar atrophy as a neuroradiological finding has been formerly discussed [2]. In association with a careful initial clinical evaluation, the characterization of cerebellar atrophy and/or hypoplasia by MRI is currently used to prioritize specialized investigations and potential diagnosis [1, 3].

The most frequent causes of early-onset cerebellar atrophy include mitochondrial disorders, neuronal ceroid lipofuscinosis, congenital disorders of glycosylation, ataxia telangiectasia and infantile neuroaxonal dystrophy [1]. The reason for the exquisite sensitivity of the cerebellum to defects in general cellular processes involving mitochondria, protein glycosylation or lysosomes is not known. The loss of Purkinje cells could play a central role in the pathology of many cerebellar ataxias as they are highly metabolic cells and these cells are the only out-put of the cerebellum.

This group of disorders includes an ever-increasing number of very rare conditions and currently over 169 OMIM clinical synopses are associated with cerebellar atrophy (OMIM December 2015). The overlapping clinical features of these numerous conditions often prevent a rapid and accurate clinical and genetic diagnosis and for more than half of the patients with childhood-onset cerebellar atrophy, a molecular diagnosis is not available [3].

Additionally, many of these conditions start with motor and cognitive delay or deterioration but some diagnostic criteria appear later during the developmental course of the disease. For example, in the case of *PLA2G6*-associated neurodegeneration (PLAN), brain iron accumulation is generally not detected in the early stages of the disease [4, 5].

In such situations whole exome sequencing (WES) has been able to accelerate molecular diagnosis, better delineate the clinical spectrum associated with specific genetic defects and improve patient management [6, 7].

In this study, we assessed a cohort of 18 families of Egyptian origin with childhood-onset cerebellar atrophy and developmental delay. We analyzed them for both recessive and dominant variants and successfully identified disease-causing mutations in half of the families (9/18). This study highlights the importance of searching for dominant mutations in consanguineous families and broadens the clinical spectrum associated with some cerebellar conditions, including molybdenum cofactor deficiency.

## Methods

### Subject information

We studied 18 families including 12 sporadic cases (67 %) and 16 families with reported consanguinity (89 %). For half of these families only the proband was exome sequenced and for the other half we performed trio exome sequencing. The mean age was 4.6 years (Standard deviation 3.2 years) at the time of the study. Patients had various degrees of cerebellar atrophy identified on MRI, involving mainly the vermis or the entire cerebellum. Both static and progressive conditions were included. Written informed consent was obtained from all families, and the study was approved by the ethics committee of the National Research Center in Cairo.

#### Whole exome sequencing

DNA was extracted from blood and the sequencing core facility at the Imagine Institute performed WES. Briefly, WES libraries were prepared from 3 μg of genomic DNA sheared by ultrasonication (Covaris S220 Ultrasonicator). Exome capture was performed with the 51 Mb SureSelect Human All Exon kit V5 (Agilent technologies). Sequencing of the WES libraries was carried out on a HiSeq2500 (Illumina). Paired-end reads were generated and mapped on the human genome reference using Burrows-Wheeler Aligner (BWA) [8]. The mean depth of coverage obtained for each sample was > 160x with >97 % of the exome covered at least 30x. SNP and indel calling was made using GATK tools. Families CIE3, 4, 5, 8, 11, 12, 13, 18 and 19 (*n* = 9; 50 %) were investigated by trio WES and for families CIE1, 2, 7, 9, 14, 16, 17, 21, 29 (*n* = 9; 50 %) WES was only performed for the proband.

#### Bioinformatics, databases

A variant filtering pipeline was systematically applied to narrow down the number of putative causative variants. All the possible inheritance patterns were tested. Briefly, common (>1 % minor allele frequency) variants were filtered out by using dbSNP, 1000 genomes databases and our in house exome collection, which includes more than 7000 exomes. Functional (protein-altering) alleles were prioritized versus non-functional. Potentially pathogenic variants in known disease genes were identified if flagged as damaging by polyphen2 (http://genetics.bwh.harvard.edu/pph2/), Sift (http://sift.jcvi.org/) or mutation taster (http://www.mutationtaster.org/). Remaining variants were compared with those in the public databases EXAC (http://exac.broadinstitute.org/) and EVS (http://evs.gs.washington.edu/EVS/) exome database. The presence of candidate recessive variants in homozygous intervals was checked by identifying predicted regions of SNP homozygosity from exome data with the unifiedgenotyper tool from GATK (https://www.broadinstitute.org/gatk/). In order to identify fully penetrant dominant mutation in singleton WES data we used the following method. We filtered out variants that were present in control individuals from our in house exome database and not predicted to be pathogenic by at least two prediction programs: PolyPhen, SIFT or Mutation-Taster. When a single rare variant predicted to be deleterious was observed in a single known cerebellar atrophy gene and associated with a dominant mode of inheritance, it was considered as potential candidate. When the same rare variant has been previously associated with disease, this was considered strong evidence that it was likely to be pathogenic. We validated potential de novo mutations by using Sanger sequencing on patients and parents DNA. However considering the large number of variants generated by this method, it is considered efficient only for the identification of mutations in known disease genes.

## Results

**Fig. 1:**
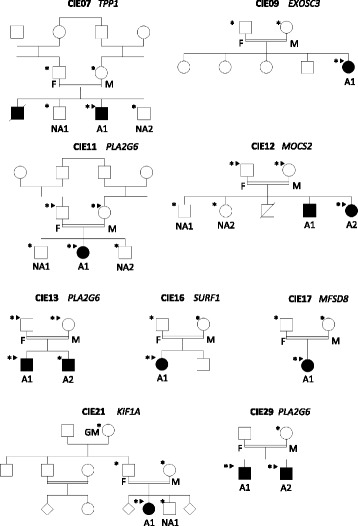
Pedigrees of families with identified disease-causing genes. A *star* indicates individuals with DNA available for segregation test and *arrowheads* point to the samples sequenced by WES

Homozygous, compound heterozygous and potential de novo mutations were investigated in all families. We identified pathological mutations in 7 genes within nine families (9/18; 50 %): a homozygous *TPP1* mutation c.790C > T (NM_000391.3) p.Gln264* in family CIE7; a homozygous *EXOSC3* mutation c.395A > C (NM_016042.3) p.Asp132Ala in CIE9; a homozygous *PLA2G6* c.2070_2072del (NM_003560.2) p.Val691del mutation in families CIE11 and CIE13; a homozygous *MOCS2* c.3G > A (NM_176806.3) p.Met1? mutation in CIE12; a homozygous *SURF1* mutation c.237G > A (NM_003172.3) p.Trp79* in CIE16; a homozygous *MFSD8* c.1213C > T (NM_152778.2) p.Gln405* mutation in family CIE17; a de novo *KIF1A* mutation c.173C > T (NM_001244008.1) p.Ser58Leu in family CIE21 and a homozygous *PLA2G6* mutation c.1613G > A (NM_003560.2) p.Arg538His in family CIE29. Mutation segregation was checked by Sanger sequencing in all available samples (indicated by a star on Fig. 1).

**Fig. 2:**
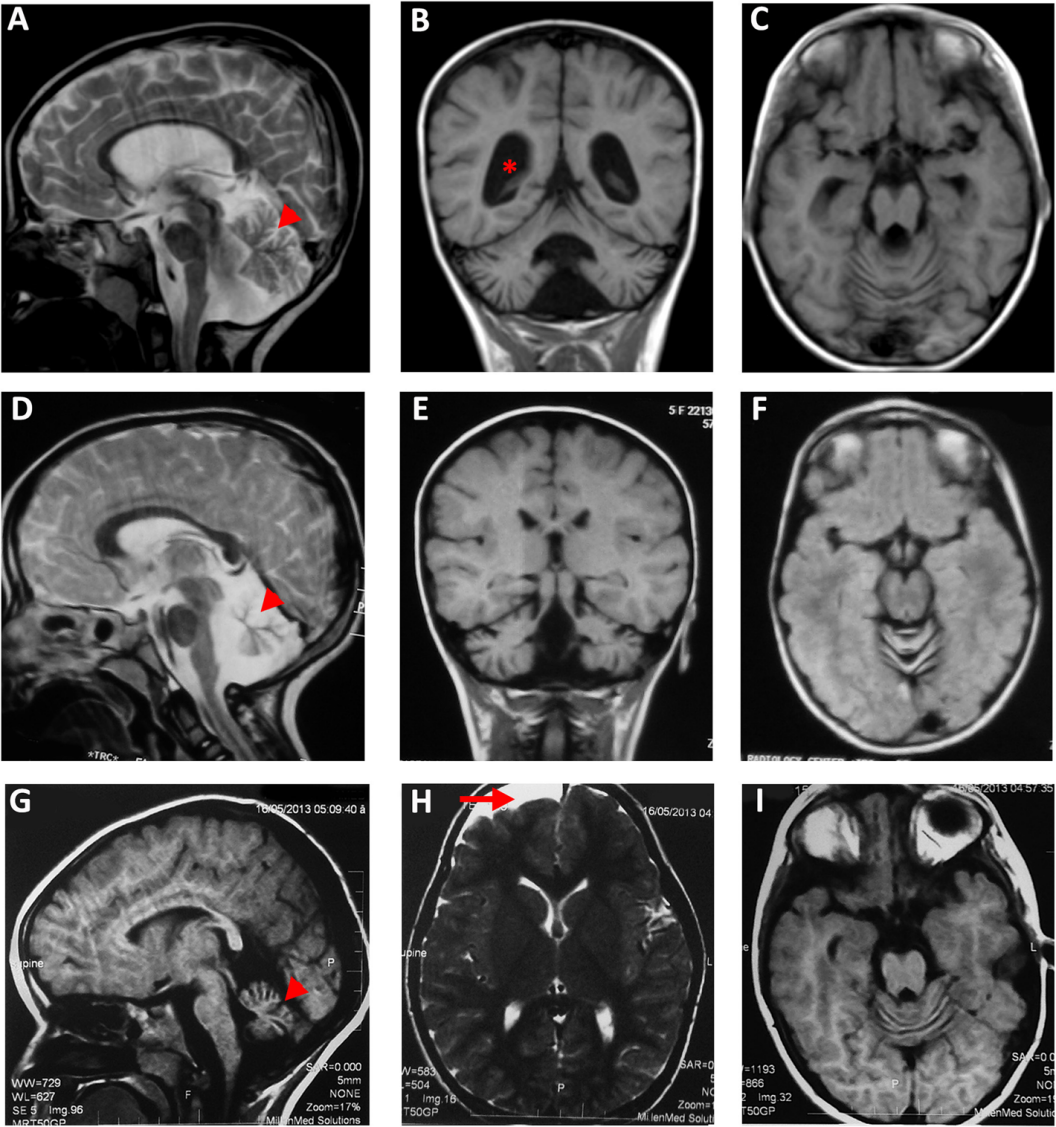
Brain MRI scans from families CIE17, CIE21 and CIE12. **a**-**c** MRIs of case CIE17-A1 (*MFSD8* mutation) at 3 years. **a** Midline Sagittal T2-weighted image demonstrating enlarged cerebellar folia (*arrow head*) and thin corpus callosum. Ventricular system dilatation (*star*) is visible on coronal T1 image (**b**). **d**-**f** MRIs of patient CIE-21-A1 (*KIF1A* mutation) at 5 years showing cerebellar atrophy (*arrowhead*) on midline sagittal T2 (**d**), coronal T1 (**e**) and axial T1 (**f**) images. **g**-**i** MRIs of patient CIE12-A2 (*MOCS2* mutation) with evidence of cerebellar vermis atrophy (*arrowhead*) on sagittal T1 image (**g**) and Right frontal arachnoid cyst (*red arrow*) visible on axial T2 scan (**h**)

Clinical findings in the affected individuals with molecular diagnosis are summarized in Table 1. Previously described mutations were identified in six genes *TPP1*, *EXOSC3*, *PLA2G6*, *MOCS2* and *SURF1* and *KIF1A*. The *PLA2G6* gene was the most frequently mutated gene and the c.2070_2072del mutation was identified in two families in our WES data. The identification of this recurrent mutation during the course of the project, led us to implement Sanger sequencing of this specific allele and only samples negative for this prescreen where submitted to WES. Using this method, we could identify a third family with the same c.2070_2072del mutation. In total, this 3 bp deletions accounts for 15 % of all the families tested in this study. The identification of *MFSD8* mutations in family CIE17 was consistent with an early onset of ceroid lipofuscinosis, neuronal 7 (CLN7) associated with cortical and cerebellar atrophy and an enlargement of the cerebral ventricles in the proband (Fig. 2a-c), a phenotype that usually manifests between 2 and 6 years old [9]. In family CIE21, we identified a recurrent de novo mutation in the *KIF1A* gene. This mutation is predicted to disrupt some hydrogen bonds in the ATP binding domain (Additional file 1: Figure S1) and results in cerebellar atrophy (Fig. 2d-f) and epilepsy (Table 1). In family CIE12, we found biallelic mutations in the initiation codon of the MOCS2A open reading frame of the bicistronic *MOCS2* gene. Mutations in this gene cause Molybdenum Cofactor (MoCo) deficiency, a severe neonatal inborn error of metabolism usually associated with subcortical multicystic lesions and death during early childhood stage [10]. Patient CIE12-A2 was born to consanguineous parents and was affected since birth. At 3.8 years, she presented with delayed speech and delayed walking. She had wide based gait, hyperreflexia and hypertonia with the left side of her body being more affected than the right. The mother had an uncomplicated pregnancy although she was exposed to X-Rays when she was one month pregnant. This patient has two unaffected siblings, one deceased brother and an older brother who suffered from global development delay since birth and mild cortical brain atrophy on MRI associated with abnormal EEG. At 6.0 years, patient CIE12-A2 appeared to improve gradually. Despite some residual unsteadiness, she can walk, read and memorize short sentences. Her initial investigations included CT and MRI, which revealed right frontal arachnoid cyst, mild right frontal lobe brain atrophy and cerebellar atrophy (Fig. 2g-i). A follow up CT two years latter indicated that the arachnoid cyst had slightly resolved. Her EEG revealed left fronto-temporal epileptogenic dysfunction. The follow up EEGs showed improvement in the epileptogenic bursts probably as a result of the antiepileptic therapy she is receiving. The molecular diagnosis was supported by the blood tests results, consistent with Molybdenum Cofactor deficiency. Plasma S-Sulphocysteine level was 309 micromol/mmol (Control value <10) and Plasma Xanthine 1291 micromol/mmol (Control value <40). She is currently receiving sodium valproate (30mh/kg), Levitiracetam (20 mg/kg), omega 3 supplements, and IM (intra-muscular) B complex.

**Table 1:** Clinical presentation, laboratory investigation and exome sequencing result for 10 patients with undiagnosed cerebellar atrophy

	CIE7-A1	CIE9-A1	CIE11-A1	CIE12-A2	CIE13-A1/A2	CIE16-A1	CIE17-A1	CIE-21-A1	CIE-29-A1
Gender	M	F	F	F	M	F	F	F	M
Gene mutated	*TPP1*	*EXOSC3*	*PLA2G6*	*MOCS2*	*PLA2G6*	*SURF1*	*MFSD8*	*KIF1A*	*PLA2G6*
Genbank reference	NM_000391.3	NM_016042.3	NM_003560.2	NM_176806.3	NM_003560.2	NM_003172.3	NM_152778.2	NM_001244008.1	NM_003560.2
Mutation cDNA level	c.790C > T	c.395A > C	c.2070_2072del	c.3G > A	c.2070_2072del	c.237G > A	c.1213C > T	c.173C > T	c.1613G > A
Mutation protein level	p.Q264*	p.D132A	p.V691del	p.M1?	p.V691del	p.W79*	p.Q405*	p.S58L	p.R538H
Final diagnosis	CLN2 Late infantile	Mild PCH type 1B	PLAN/INAD	Mild MoCo deficiency	PLAN/INAD	LEIGH SYNDROME	CLN7	AD ID	PLAN/INAD
Age of onset (years.months)	3.1	1	0.9	Neonatal	1.6/1.0	1	0.6	2.0	1.5
Last follow-up (years.months)	4.6	1.9	3.0	6.0	4.0/2.0	4.7	3.6	5.0	3.6
Initial symptom	Convulsions	Developmental delay	Convulsions	Developmental delay	Convulsions	Gait disturbance	Developmental delay	Gait disturbance	Developmental regression
Development									
Developmental delay	+	+	+	+	+	+	+	+	+
Motor developmental delay	+	+	+	+	+/++	+	+	+	+
Social development delay	+	+	+	+	+/++	+	++ (autistic features)	+	+
Progressive condition	+	-	Mildy progressive	-	+	+	+	-	+
Seizures									
Description	Focal epileptic activity	GTC	Focal Right-temporal discharges, GTC	Left fronto-temporal epileptogenic dysfunction	Focal, GTC	GTC	Right frontal epileptogenic focus, Akinetic fits	Right-temporal activity, intractable epilepsy	GTC
Neurological Findings									
Hypotonia	+	+	+	- (Hypertonia)	+	+	+	+	+
Nystagmus	-	+	+	-	+	+	-	-	+
Wide-based, staggering gait	+	+	Enable to walk	Wide based gait	+	Tetubation, ataxia	+	Wide-based, staggering gait	Tetubation,ataxia
Peripheral neuropathy	-	+	+	-	+	+	-	+	+
MRI									
Cerebellum:Hypoplasia/Progressive or fixed Atrophy	Atrophy/hypoplasia	Atrophy	Atrophy,hypoplasia, dilated cisterna magna	Atrophy	Atrophy	Atrophy + abnormal signal intensity	Atrophy	Atrophy	Atrophy
Brainstem	-	-	-	-	-	Abnormal signal intensity as well as in BG	-	-	-
Cerebral cortex	Mild cortical atrophy	-	-	Right frontal arachnoid cyst, mild frontal lobe atrophy	Mild cortical atrophy/-	-	Atrophy	-	-
Ventricular system	-	-	Dilated	-	Moderate dilatation	-	Mild dilatation	-	-
Facial dysmorphism	-	Squint	-	Mild dysmorphism	-	-	-	-	-
Ophtalmologic finding	Fundus exam: Macular lesion	-	-	-	-	-	-	-	-
Relevant metabolic result				High Plasma S-Sulphocysteine level and high plamsa Xhantine level (see text)		Plasma lactate in the normal range	Blood ammonia: 62.2 μmol/L (normal 15–45); blood lactate: 20.6 mmol/L (normal = 0.5-2.2)		

*Abbreviations: GTC* generalized tonic-clonic, *PCH* pontocerebellar hypoplasia, *PLAN* PLA2G6-associated neurodegeneration, *INAD* infantile neuroaxonal dystrophy*, MoCo* Molybdenum cofactor, *CLN* Ceroid lipofuscinosis neuronal, *AD* autosomal dominant, *ID* intellectual disability, *BG* basal ganglia

## Discussion

In this study, we explored sporadic and multiplex Egyptian families with early-onset cerebellar atrophy associated with developmental delay by using high-coverage whole exome sequencing. Our analysis included the investigation of recessive and dominant variants and we identified pathogenic mutations in 50 % of the 18 families studied. Previous reports that focused on sporadic and inherited cerebellar atrophies [11] or ataxias [12] and obtained comparable success rates of 39.1 and 41 % respectively, in the identification of pathological mutations.

The absences of diagnosis for half of the cohort could be explain with the following reasons. Despite high depth of sequencing (mean depth of coverage > 160x), exome capture does not provide complete coverage of all coding regions of the genome, particularly those with GC-rich regions. Moreover, large genomic rearrangements and trinucleotide repeat sequences are not reliably detected from exome-capture data. It is also possible that some causal variants will reside within non-coding regulatory regions. Some of these issues will be resolved by whole genome sequencing, although not without substantial additional cost and bioinformatics analyses. However it is likely that mutation in yet unknown cerebellar atrophy genes contribute significantly to these diseases. In this study, half of the patients were explored by using trio WES and the other half by singletons WES. Trio WES did not improve the diagnosis rate but considerably decreased the number of Sanger cosegregation analyses. It impacted specially de novo mutations analysis but singleton WES did not prevent de novo mutation identification as illustrated with the *KIF1A* gene mutation. One of the clear advantages of trio WES is its ability to point to a limited number of variants located in novel candidate disease-causing genes when mutations located in known disease gene are absent. The identification of *KIF1A* de novo mutation highlights the importance of considering not only recessive inheritance patterns when analyzing consanguineous exomes despite the presence of extensive regions of homozygosity (data not shown). This finding identifies p.Ser58Leu as a recurrent *KIF1A* mutation [13] and indicates that cerebellar atrophy is the main MRI feature associated with this allele. Several typical symptoms of infantile-onset *PLA2G6*-associated neurodegeneration (PLAN) are not always observed before 4 years, such as optic atrophy, electroencephalogram fast rhythms and amyotrophy [4], making very early diagnosis more difficult. *PLA2G6* is the most frequently mutated gene in this cohort; 3 families were identified with the same homozygous mutation previously described as a founder mutation in Mediterranean countries [4]. This observation suggests that the p.Val691del allele is an especially common cause of PLAN in the Egyptian population. Among other clinical symptoms, patients with this mutation shared the association of development delay or regression with cerebellar atrophy, epilepsy and nystagmus. Mutations in the *MOCS2* genes cause MoCo deficiency type B, which is currently untreatable [14]. These mutations can occur in one of the two open-reading frames (i.e., MOCS2A and MOCS2B) of this bi-cistronic gene and are generally associated with untreatable seizures, multiple cystic cavities on MRI and death at early age [10]. Milder cases have been reported to be associated with a hypomorphic allele [15] or mutations involving a non-constitutively spliced exon [16]. Strikingly, the p.Met1? mutation identified in MOCS2A has been tested in vitro and shown to abolish translation [17]. This result highlights the existence of mild presentations of MoCo syndrome, potentially detectable with plasma sulfite and xanthine screening of patients with undiagnosed cerebellar atrophy, developmental delay and isolated arachnoid cyst.

## Conclusions

Our study emphasizes the benefits of whole exome sequencing to efficiently diagnose early-onset cerebellar atrophy defects, to better delineate the clinical spectrum associated with these disorders and to open the way for the identification of new disease genes.

## Ethics approval and consent to participate and for publication

Written informed consent was obtained from all families, and the study was approved by the ethics committee of the National Research Center in Cairo.

## Additional file

Additional file 1: Figure S1. Modeling of the p.S58L mutation using the crystal structure of the motor domain of human KIF1A (PDB number 1VFV). The image was generated using PyMOL (http://www.pymol.org). The side-chain of Ser58 is involved in inferred hydrogen bonds (arrow) with other amino-acids located in the ATP-binding pocket (A). These interactions are predicted to be disrupted by the mutation (B) including the hydrogen bonds with the highly conserved Arginine 11 that interacts with ATP through a molecule of water. (JPG 499 kb)

## Abbreviations

MoCo: molybdenum cofactor
PLAN: *PLA2G6*-associated neurodegeneration
WES: whole exome sequencing
