# Supplementary figures and images for: Utility of whole exome sequencing for the early diagnosis of pediatric-onset cerebellar atrophy associated with developmental delay in an inbred population
Source: Orphanet J Rare Dis. 2016 May 4;11:57. doi: 10.1186/s13023-016-0436-9 (PMC4855324; doi:10.1186/s13023-016-0436-9)

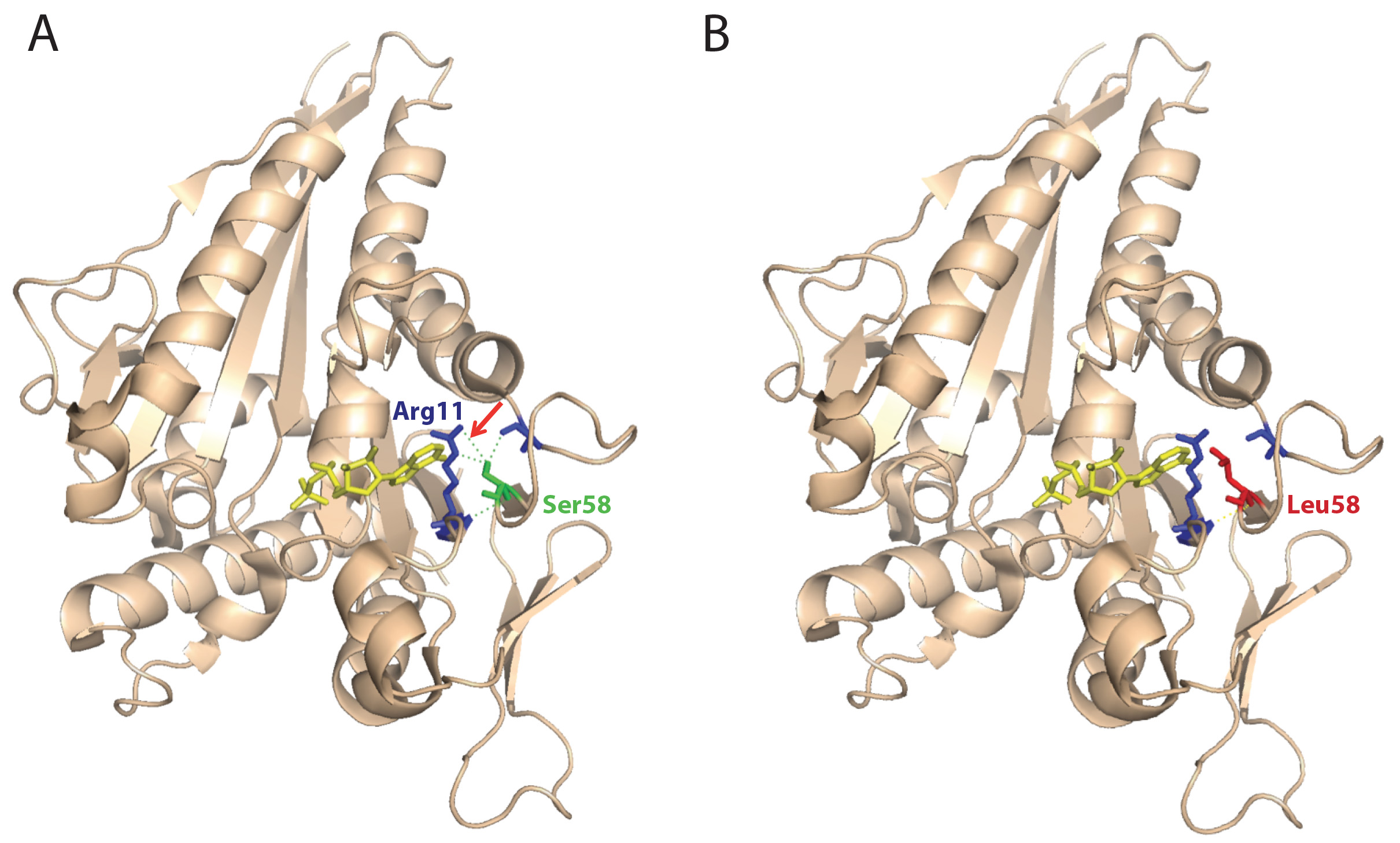

Supplement: Additional file 1: Figure S1. — Modeling of the p.S58L mutation using the crystal structure of the motor domain of human KIF1A (PDB number 1VFV). The image was generated using PyMOL (http://www.pymol.org). The side-chain of Ser58 is involved in inferred hydrogen bonds (arrow) with other amino-acids located in the ATP-binding pocket (A). These interactions are predicted to be disrupted by the mutation (B) including the hydrogen bonds with the highly conserved Arginine 11 that interacts with ATP through a molecule of water. (JPG 499 kb) [file 13023_2016_436_MOESM1_ESM.jpg]
